# Community health workers identify children requiring health center admission in Northern Uganda: prehospital risk prediction using vital signs and advanced point-of-care tests

**DOI:** 10.1080/16549716.2025.2519704

**Published:** 2025-06-26

**Authors:** Daniel Ebbs, Olanya Denish, Felix Bongomin, Arjun Chandna, Fathima Haseefa, Michael Canarie, Michael Cappello

**Affiliations:** a Department of Pediatrics, Yale University School of Medicine, New Haven, CT, USA; b Northern Uganda Medical Mission, Pader, Uganda; c School of Health Sciences, Kampala International University, Kampala, Uganda; d Department of Medical Microbiology and Immunology, Faculty of Medicine, Gulu University, Gulu, Uganda; e Department of Internal Medicine, Gulu Regional Referral Hospital, Gulu, Uganda; f Cambodia Oxford Medical Research Unit, Angkor Hospital for Children, Siem Reap, Cambodia; g Centre for Tropical Medicine and Global Health, Nuffield Department of Medicine, University of Oxford, Oxford, UK; h Department of Emergency Medicine, Creighton University, Phoenix, AZ, USA; i Department of Epidemiology of Microbial Diseases, Yale School of Public Health, New Haven, CT, USA

**Keywords:** Community health worker, village health teams, point-of-care tests, seriously ill children, rapid diagnostic tests, prehospital prediction, prehospital point-of-care

## Abstract

**Background:**

Over five million children die annually from preventable and treatable illnesses. Most of these deaths occur in sub-Saharan Africa, predominantly in socioeconomically deprived regions. With nearly half of pediatric mortality occurring at the community level, serious illnesses must be detected early in the prehospital setting. The purpose of this 18-month, prospective, observational pilot study was to introduce the first use of the proinflammatory biomarker, CRP, in the prehospital setting to community health workers and to develop a prehospital predictive model to identify sick children requiring health center admission.

**Methods:**

We recruited 636 children presenting to one of four community health worker teams who completed a prehospital evaluation and referred each child to the closest health center. The primary outcome for this study was admission at the health center for more than 24 h. We used logistic regression to quantify the area under the receiver operating characteristic curve (AUC).

**Results:**

We found poor discrimination of danger signs and CRP, with AUCs of 0.55 (95% CI 0.52–0.57) and 0.52 (95% CI 0.47–0.57), respectively. A model comprising vital signs demonstrated superior discrimination, with an AUC of 0.66 (95% CI 0.62–0.71), which improved with the addition of danger signs (AUC 0.69; 95% CI 0.64–0.73), and when restricted to children who tested negative for malaria (*n* = 327; AUC 0.71; 95% CI 0.65–0.77).

**Conclusions:**

We demonstrate that performing advanced point-of-care tests is feasible in resource-limited community settings and present one of the first prehospital prediction models developed with community health workers.

## Paper Context

- **Main Findings:** Community health workers develop the first prehospital pediatric risk prediction model with advanced point-of-care testing.
- **Added knowledge:** Advanced point-of-care testing can be conducted by community health workers in prehospital settings; current standard of care for identifying seriously ill children has poor discrimination for hospital admission.
- **Impact:** A prehospital vital signs model demonstrates superior discrimination and can be expanded to create the first evidence-based risk prediction tool to identify seriously ill children at the community level.

## Background

Since the early 1990s, pediatric mortality rates in low- and middle-income countries (LMICs) have decreased by over 50% [1,2]. This reduction, however, is not uniform among pediatric age groups and regions across the globe [1,2]. Sub-Saharan Africa, for example, has the highest risk of childhood death and LMICs in this region lag behind others in annual mortality rate reductions [1,2]. This is partially attributed to limited socioeconomic development and lack of affordable and quality health services, particularly in rural locations [1–3]. Although the average under-five mortality rate is approximately 74/1000 across Sub-Saharan Africa, it is common to find rates as high as 1/10 in the lower socioeconomic sectors of each country [1–3]. Uganda is no different. The Uganda Demographic and Health Survey highlights how pediatric mortality rates are linked to socioeconomic status, access to basic health services, and education [3]. Given these disparities, tailored approaches are required to reduce mortality.

One solution to combatting childhood mortality is to empower community health workers (CHWs) to serve as access points for early triage and treatment of common local health problems [4–6]. In the late 1990s, the World Health Organization (WHO) developed a training framework, the Integrated Management of Childhood Illnesses (IMCI), to disseminate standards for focused facility-based pediatric care in limited resource settings [7–9]. No more than a decade later, this expanded to the prehospital setting for CHWs through a program called integrated Community Case Management (iCCM) [7–9]. iCCM provides diagnostic and treatment algorithms for CHWs to support recognition and treatment for three of the most common community illnesses: malaria, diarrhea, and pneumonia in the prehospital setting [7–9]. Introduction of iCCM among vast networks of CHWs has increased capacity for further training [8,9]. For example, iCCM utilizes rapid diagnostic tests to evaluate for malaria, meaning that many CHWs are now competent to complete basic point-of-care tests (POCTs) [7–9].

Over the past decade, POCTs have transformed how patients are triaged in resource-abundant health systems [10–14]. Proinflammatory biomarkers such as C-Reactive Protein (CRP) and Procalcitonin (PCT), once novel with uncertain clinical significance, have become the standard of care for many presenting medical conditions [10–14]. CRP, unlike other inflammatory biomarkers, can be administered and sustained in a prehospital setting due to its analyzer portability, capillary blood sampling, and maintenance without electricity using solar panels. Aligned with the advancement of medical biotechnology, several hundred new potential biomarkers have been developed in search of identifying patients at high risk for poor outcomes [12,15]. Diagnostic accuracy among many of these seem promising; however, none has been evaluated in the prehospital setting. This is particularly important as a substantial proportion of preventable childhood deaths occur outside of hospital [16]. The impact of such a POCT in this region and setting remains unknown and may have significant clinical benefit.

In addition to the limited use and evidence for prehospital POCTs for identifying serious illnesses, no predictive score has been evaluated for children in the prehospital setting. Many predictive scores, such as the Lambarene Organ Dysfunction Score (LODS), Signs of Inflammation in Children that Kill (SICK), or the Pediatric Early Death Index for Africa (PEDIA), demonstrate excellent diagnostic accuracy for mortality risk among rural health clinics in limited resource settings [17–19]. These scores have demonstrated similar prognostic accuracy for children presenting to a hospital in Eastern Uganda [20]. However, no score or algorithm has been evaluated in the prehospital setting utilizing CHWs.

The purpose of this 18-month, prospective, observational pilot study was to introduce the first use of the proinflammatory biomarker, CRP, in the prehospital setting by CHWs and to develop a prehospital predictive model to identify children requiring health center admission.

## Methods

This study is reported in accordance with the Transparent Reporting of a multivariable prediction model for Individual Prognosis Or Diagnosis (TRIPOD) guidelines [21].

### Ethics statement

Ethics approval was granted by Mulago Hospital Research and Ethics Committee in Uganda (MHREC 215), in addition to Yale University School of Medicine in the United States. A letter of support was granted by the Uganda Ministry of Health for this research. Formal written consent was obtained from all participants in this study and was available in both Acholi and English languages. For participants less than 18 years of age, written informed consent was obtained from their parent or guardian.

### Study population and source of data

This 18-month prospective observational study was conducted from January 2022 to July 2023 among four rural villages in Pader District, Northern Uganda. Each rural village has an appointed Village Health Team (VHT), similar to CHWs, who are members of each community and act as the first point of access for community health concerns and emergency triage. VHTs in this study, in addition to those in surrounding districts, have been trained to measure vital signs through a community-based training program called the Laro Kwo Project (LKP), which translates to ‘saving lives’, in Acholi. LKP has been previously described [22]. Briefly, VHTs are equipped with medical equipment to evaluate prehospital vital signs and undergo competency examinations on medical skills annually. VHT training includes pediatric focused triage where respiratory rate, pulse rate, and axillary temperature are recorded prior to referral of all children with non-traumatic complaints to a local health center for further assessment. Currently, only children with injuries requiring minor first aid are managed by the VHT in a community setting. The four VHTs in this study were selected based on [1] previous appointment from their village and enrollment in the LKP [2], repeated competency evaluation on skills ≥85%, and [3] proximity (<30-min travel time) to the referral health center, Northern Uganda Medical Mission Clinic (NUMEM) in Pader. Achieving a score of 85% or above is the current benchmark to pass any LKP skills evaluation, and this score was determined from expert consensus among a team of Ugandan and US physicians.

### Participants

#### VHTs

Four VHTs were selected and consented based on the above criteria. With enrollment, each VHT agreed to complete CRP POCT competency examinations immediately after initial training and at two, six, and 12 months into the study; this timeline for repeated skills examination is the standard as agreed upon by U.S. and Ugandan medical directors for the Laro Kwo Project. Prior to training, a checklist was developed by the NUMEM team that includes all core steps in completing the CRP POCT (supplemental 1). This team included the NUMEM research director and a laboratory technician. Each step was graded with 0 or 1 point, and a score of ≥85% was required to pass the skill. A process was agreed for allowing one retake for competency examination the next day if the score was below 85%. All VHTs enrolled were required to maintain a score competency of ≥85%.

#### Children

After demonstrating competency in CRP POCT testing, each VHT began screening children aged 2–59 months presenting for any non-traumatic complaint for recruitment into the study. The majority of pediatric VHT presentations in Pader District are for fever (50–65%) and/or increased work of breathing (35–45%). The current standard of care for VHT practice for non-traumatic complaints in the LKP is to [1] complete a history and evaluate the patient by measuring vital signs [2], administer paracetamol for fever, and [3] refer the patient to the closest clinic for further evaluation. LKP VHTs have been trained to follow iCCM recommendations; however, inconsistent availability of antimicrobial drugs has limited their application of these guidelines.

If consent were provided by the guardian, each participating child was referred to NUMEM health center for further evaluation and carried the VHT prehospital examination results, which included their vital signs, a CRP value, and the presence or absence of WHO iCCM danger signs (seizures, altered mental state, inability to drink or breastfeed, and persistent vomiting of oral intake). Part of the enrollment included the requirement to agree to immediately travel to the NUMEM health center. Children were ineligible to enroll in this study if a parental or guardian was not present and consented, if the child was outside the study age range, or if the family could not travel to the health center after a VHT referral. NUMEM employs advanced practitioners 24 h a day and is run by a team of Ugandan physicians. At NUMEM, each participant was triaged, examined, and received a CBC and blood smear for malaria. Providers at NUMEM were blinded to prehospital CRP value and determined admission based on their own physical examination and vital signs.

### Outcome

The primary outcome for this study was admission to the NUMEM health center, which was defined as remaining as an inpatient in the facility for >24 h. NUMEM providers were unaware of the outcome definition for this study. Admission duration for each enrolled patient was determined by chart-review by the research coordinator.

### Predictors

Predictors evaluated for admission included the presence of a WHO iCCM danger sign, sepsis as defined by Goldstein’s criteria [23], malaria infection, CRP, and a multivariable prediction model comprising age, heart rate, respiratory rate, and axillary temperature (Table 1). Predictors were chosen to limit adding more than one new advanced variable to VHTs training (CRP); all other variables are collected by VHTs as standard of care. Vital signs, including heart rate, axillary temperature, and respiratory rate, CRP, and presence of danger signs were assessed at initial presentation to the VHT. VHTs completed a vital signs examination using a stopwatch or sand timer to count both respiratory and heart rates over a 60-s interval. Axillary temperature was recorded using a digital thermometer. CRP was measured via capillary puncture with the Inclix POCT immunoassay analyzer at initial presentation. The Inclix POCT immunoassay analyzer was connected to solar panels due to unreliable electricity. WBC was evaluated at arrival to NUMEM and used to determine the presence of sepsis as per Goldstein’s criteria [23]. As standard of care for all pediatric referrals, a malaria blood smear was collected upon arrival to NUMEM health center.

**Table 1.** All children evaluated by CHW and enrolled in the study.

		Admission >24 h		
Characteristic	Overall, *N* = 636^a^	No, *N* = 194^a^	Yes, *N* = 442^a^	*p*-value^b^
Age (months)	12.0 (9.0, 36.0)	24.0 (12.0, 36.0)	12.0 (9.0, 24.0)	<0.001
Heart rate (bpm)	120.0 (104.0, 148.0)	110.0 (100.0, 131.5)	122.5 (105.0, 150.0)	<0.001
Respiratory rate (bpm)	37.0 (32.0, 42.0)	37.0 (32.0, 42.0)	38.0 (32.0, 42.0)	0.70
Axillary temperature (°C)	37.6 (36.7, 38.6)	37.4 (36.6, 38.2)	37.8 (36.9, 38.6)	0.016
White cell count (×10^9^ cells/L)	8.9 (6.9, 11.6)	8.5 (6.7, 10.8)	9.0 (7.0, 12.0)	0.036
WHO iCCM danger sign	72/636 (11%)	10/194 (5.2%)	62/442 (14%)	0.001
Sepsis	278/614 (45%)	81/186 (44%)	197/428 (46%)	0.60
Malaria blood smear positive	309/636 (49%)	98/194 (51%)	211/442 (48%)	0.50
C-reactive protein (mg/L)	18.0 (2.5, 70.9)	15.7 (2.5, 59.9)	19.6 (2.5, 73.7)	0.40
C-reactive protein >40mg/L	223/636 (35%)	58/194 (30%)	165/442 (37%)	0.070

Note: Missing Data Total = () per Characteristic: Age (35), Axillary Temp [11], White Cell Count [10], Sepsis [22].

^a^Median (IQR); n/N (%).

^b^Wilcoxon rank sum test; Pearson’s Chi-squared test.

### Sample size

The monthly average number of pediatric referrals in this age group varies due to season and the prevalence of malaria. From LKP data over the last several years, we estimated the four advanced VHTs in Pader District to average approximately 6–10 referrals per month and a total number of approximately 720 referrals over 18 months. Repository data from the three local referral clinics indicated that approximately 45–55% of children less than 5 years of age who present with fever and/or increased work of breathing are admitted for more than 24 h. Assuming a c-statistic of 0.70, a shrinkage factor of 0.9, and an outcome prevalence of 0.50, the minimum sample size required to build a multivariable prediction model comprising five parameters was estimated to be 385, suggesting that the study would be adequately powered if it recruited for 18 months [24].

### Missing data

Of the 636 children, 580 (91.2%; 580/636) had complete data on all baseline predictor variables. Age had the highest proportion of missing data (5.5%; 35/636; supplemental 2). Median imputation grouped by outcome status was used to address missing data [25]. Eighteen children (2.8%) were referred; however, they did not arrive at the health center.

### Statistical analysis

The ability of each predictor (WHO iCCM danger signs, sepsis, or CRP) to discriminate children who would require admission at NUMEM was assessed using logistic regression to quantify the area under the receiver operating characteristic curve (AUC; R package: *pROC*) [26]. Discrimination of each predictor was compared using the DeLong test [27].

Prior to building the vital signs model, the relationship between each predictor and the outcome was explored using locally weighted scatterplot smoothing (LOWESS) to identify non-linear patterns [28]. Logistic regression was used to derive the model. Stratum-specific odds ratios and likelihood ratio tests (LRTs) were used to test for interaction between age and each of heart rate and respiratory rate, as well as between temperature and each of heart rate and respiratory rate. The added value of CRP and danger signs to the vital signs model was explored by comparing the discrimination of a model containing vital signs only to models containing these additional parameters [27]. A subgroup analysis was performed stratified by a child’s malaria status.

All analyses were performed in R, version 4.2.2 [29].

## Results

All VHTs completed competency examinations immediately after initial training and at two, six, and 12 months into the study. All VHTs scored >85% on each exam; no additional retakes or training were required.

Six-hundred and thirty-six children aged 2–59 months were enrolled over 18 months. The maximum time between VHT assessment and arrival at NUMEM health center was 3 h. Primary outcome status was available for all children: 69% (442/636) were admitted at NUMEM for more than 24 h (Table 1). Median age was 12 months (inter-quartile range [IQR] = 9–36 months), with children who required admission to NUMEM being significantly younger (12 vs 24 months; p < 0.001). Half of the children enrolled (49%; 309/636) had malaria parasites identified on a blood smear. Median CRP concentration was 18 mg/L (IQR 2.5 to 70.9 mg/L) and did not differ between children who were admitted to NUMEM and those who were managed in the community (15.7 vs. 19.6 mg/L; *p* = 0.40). Seventy-two children (11%; 72/636) had at least one WHO iCCM danger sign and this proportion was higher in children who required admission to NUMEM (14% [62/442] vs. 5.2% [10/194]; *p* = 0.001).

### Poor discrimination of danger signs, sepsis, and CRP for identification of children requiring admission

The odds of admission were significantly greater in children who had at least one WHO iCCM danger sign compared to those who had no danger signs (odds ratio [OR] = 3.00; 95% CI = 1.57 to 6.35; *p* = 0.002). There was no difference in the odds of admission for children with higher CRP concentrations (OR = 1.00; 95% CI = 1.00 to 1.00; *p* = 0.39) or in those who did and did not meet Goldstein’s criteria for sepsis (OR = 1.11; 95% CI = 0.79 to 1.56; *p* = 0.57). With respect to predictive performance, the presence of an WHO iCCM danger sign(s), the current prehospital standard of care for identifying children at risk of serious illness requiring health center admission, demonstrated poor discrimination for predicting admission, with an AUC of 0.55 (95% confidence interval [CI] = 0.52 to 0.57). Similarly, the presence of sepsis (as defined by Goldstein’s criteria) was equally poorly discriminative (AUC = 0.51; 95% CI = 0.47 to 0.55), as was CRP concentration (AUC = 0.52; 95% CI = 0.47 to 0.57).

### Improved performance of a vital signs model

**Figure 1.**
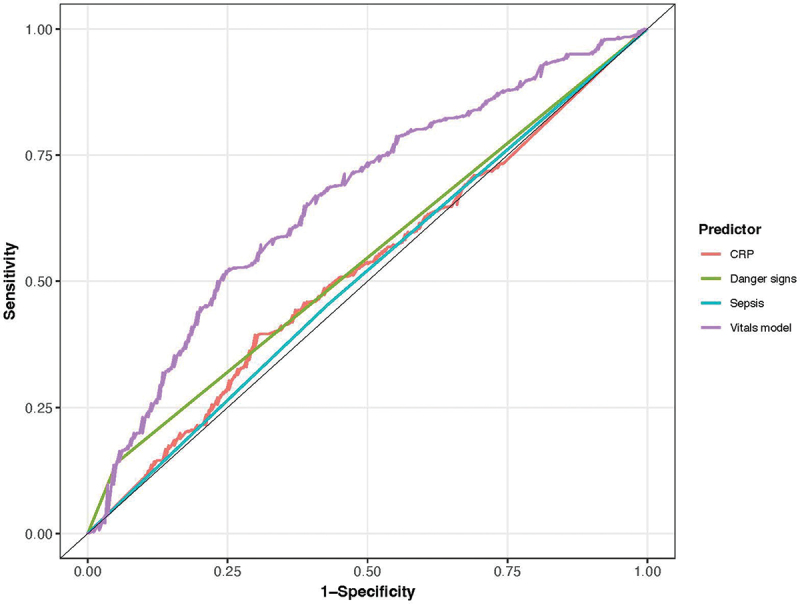
Areas under the receiver operating characteristic curves (AUCs) for predicting admission at NUMEM. AUC for CRP = 0.52 (95% CI = 0.47 to 0.57); iCCM danger sign = 0.55 (95% CI = 0.52 to 0.57); sepsis = 0.51 (95% CI = 0.47 to 0.55); and the vital signs model = 0.66 (95% CI = 0.62 to 0.71).

Relationships between each predictor and the primary outcome are illustrated (supplemental 3). Gross deviations from linearity were not observed. There was no evidence of interaction between heart rate and age (LRT = 0.63; *p* = 0.43) or temperature (LRT = 0.60; *p* = 0.44), or between respiratory rate and age (LRT = 2.27; *p* = 0.13) or temperature (LRT = 0.00; *p* = 0.97). Discrimination of the vital signs model to identify children who would require admission was 0.66 (95% CI = 0.62 to 0.71; Figure 1) and significantly better than the predictive ability of WHO iCCM danger signs (AUC 0.66 vs. 0.55; *p* < 0.001). Addition of CRP to the vital signs model did not improve performance (AUC = 0.67; 95% CI = 0.62 to 0.71; *p* = 0.79). Addition of danger signs to the vital signs model resulted in a small improvement in performance (AUC = 0.69; 95% CI = 0.64 to 0.73; *p* = 0.02).

### Superior performance of the vital signs model in children with non-malarial illnesses

**Figure 2.**
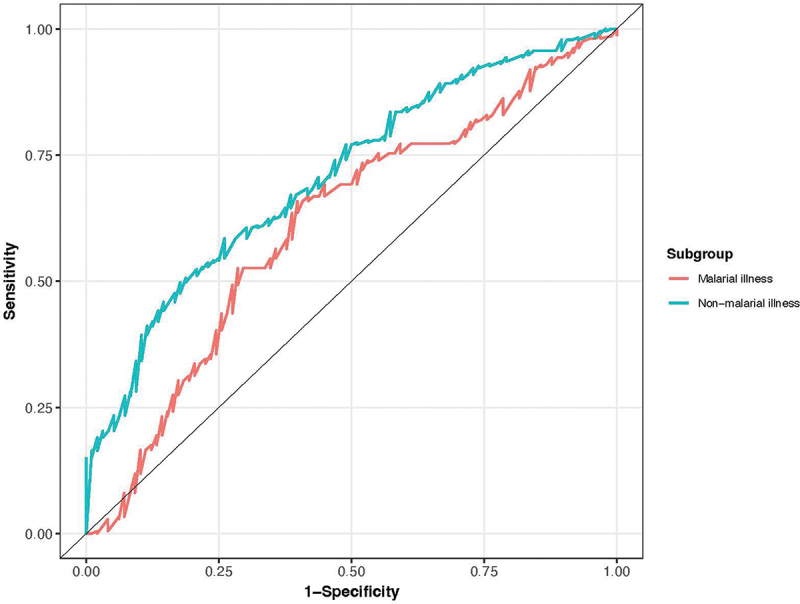
Areas under the receiver operating characteristic curves (AUC) for the vital signs model in children with and without malaria parasites on their blood smear. AUC for children with malaria parasites = 0.61 (95% CI = 0.54 to 0.68) and for children without malaria parasites = 0.71 (95% CI = 0.65 to 0.77).

Given that many CHWs have been successfully trained to use POCTs to test and treat for malaria, and that established criteria exist to support the identification of children with severe malaria requiring referral, a subgroup analysis was performed to evaluate the performance of the model in children with non-malarial illnesses. Baseline characteristics of children with non-malarial illnesses are presented in Table 2, stratified by their primary outcome status. Discrimination of the vital signs model was superior in children with non-malarial illnesses compared to those who were blood smear positive for malaria parasites (AUC = 0.71 [95% CI = 0.65 to 0.77] vs. 0.61 [95% CI = 0.54 to 0.68]; *p* = 0.04; Figure 2), with discrimination improving slightly with the addition of danger signs (AUC = 0.74; 95% CI = 0.68 to 0.79), although this difference did not reach statistical significance (AUC 0.74 vs. 0.71; *p* = 0.08).

**Table 2.** Cohort of children enrolled without malaria parasites on their blood smear.

		Admission >24 h		
Characteristic	Overall, *N* = 327^a^	No, *N* = 96^a^	Yes, *N* = 231^a^	*p*-value^b^
Age (months)	12.0 (8.0, 24.0)	24.0 (12.0, 36.0)	12.0 (7.0, 24.0)	<0.001
Heart rate (bpm)	120.0 (104.5, 145.0)	110.0 (100.0, 121.2)	120.0 (105.0, 150.0)	<0.001
Respiratory rate (bpm)	38.0 (32.0, 44.0)	38.0 (32.0, 44.5)	38.0 (32.0, 43.0)	>0.90
Axillary temperature (°C)	37.3 (36.7, 38.3)	37.1 (36.7, 38.0)	37.5 (36.7, 38.5)	0.073
White cell count (×10^9^ cells/L)	8.5 (6.7, 11.3)	8.2 (7.0, 10.7)	8.8 (6.4, 11.5)	0.40
WHO iCCM danger sign	39/327 (12%)	4/96 (4.2%)	35/231 (15%)	0.005
Sepsis	120/312 (38%)	31/91 (34%)	89/221 (40%)	0.30
C-reactive protein (mg/L)	10.4 (2.5, 55.2)	11.2 (2.5, 60.8)	9.7 (2.5, 51.8)	0.50
C-reactive protein >40mg/L	91/327 (28%)	27/96 (28%)	64/231 (28%)	>0.9

Missing Data Total = () per Characteristic: Age [22], Axillary Temp [6], White Cell Count [6], Sepsis [15].

^a^Median (IQR); n/N (%).

^b^Wilcoxon rank sum test; Pearson’s Chi-squared test.

## Discussion

This study found that the current standard of care (presence of WHO iCCM danger signs) has poor predictive value in identifying sick children requiring health center admission in Northern Uganda. We also demonstrated that CHWs can maintain and utilize advanced POCTs with solar panels in their community, and that the POCT evaluated in this study, CRP, has poor discriminatory ability for health center admission. Although this is a pilot study, we suspect that poor predictive accuracy for CRP is due to the high prevalence of concomitant malnutrition and malaria. Furthermore, we developed the first prehospital prediction model integrating vital signs, danger signs and CRP POCT results. Our results indicate that vital signs have superior discriminatory ability for identifying children requiring health center admission and that including danger signs alongside vital signs might augment performance further. By stratifying our analyses according to a child’s malaria status, we found that the model had greater predictive value in identifying admission among children who did not have malaria parasites visible on their blood smear.

An evidence-based, prehospital risk prediction model that can be used as an assessment tool by CHWs has the potential to significantly reduce pediatric mortality in LMICs. This study demonstrates that a predictive model with vital signs and danger signs, alongside other diagnostic tools (POCTs) is feasible for development and implementation by CHWs. Although CRP POCTs demonstrated poor discriminatory ability for identifying seriously ill children in this study, the successful use of advanced POCT analyzers can guide future prehospital models that integrate an expanding profile of POCTs. For example, several other biomarkers evaluated in various LMIC contexts demonstrate promising potential for the identification of children at high risk of serious illness [15,30]. The soluble triggering receptor expressed on myeloid cells-1 (sTREM-1), for example, has demonstrated discrimination greater than 0.90 for predicting mortality among hospitalized children in Uganda [15], while Angiopoietin-2 (Ang-2) demonstrated discrimination greater than 0.80 for predicting oxygen requirement among children with WHO-defined pneumonia on the Thailand-Myanmar border. Both of these biomarkers appear to add value to simple predictive clinical models. The simultaneous advancement in biotechnology with the discovery of novel biomarkers makes this an optimal time to bring risk prediction tools to the village. Developing community-based models for research, similar to the ongoing model used in this study in Northern Uganda (LKP), can facilitate the implementation of prediction tools and ultimately reach children at risk of serious illness earlier.

There are several limitations to this study. Patients were enrolled in the same district, close to NUMEM, and by referral from only four CHWs. This lack of geographic diversity limits generalizability due to regional differences in populations and proximity to health resources. Further, this was a pilot study with a small sample size, limiting our ability to validate our model and include other outcomes relevant to predicting serious illnesses such as the requirement for transfer to a regional hospital or mortality. Although admission is a pragmatic and appropriate outcome by which to evaluate community-based triage strategies, duration of admission can be influenced by factors other than illness severity and may have introduced outcome misclassification, hampering our ability to identify useful predictors. Furthermore, although advanced providers were blinded to outcome and evaluated all patients after arrival at NUMEM, access to referral forms was available with all data except CRP. This can introduce bias to admission decision.

This study used prehospital clinical variables as predictors for model development, with possible measurement error given that CHWs are volunteers who lack professional medical training. To prevent error in vital and danger signs evaluation, CHWs selected for this study had demonstrated persistent competency with vital signs over two years. Furthermore, an objective evaluation form was used that included check boxes for each danger sign to facilitate patient questioning. Overall, training with the subsequent collection of clinical data in the village is pragmatic and real-world as it reflects the cadre of health workers who would ultimately use and benefit most from such a model. Additionally, although equipment used by CHWs, such as sand-timers and digital thermometers, has been validated by a clinical team comprising Ugandan and U.S. physicians for all CHWs in the LKP, minimal variability in results is possible. Finally, NUMEM providers were only blinded to CRP results and had access to standardized referral sheets with recorded vital and danger signs. Although history and exam were repeated at NUMEM, access to referral sheets may have introduced work-up bias and could have inflated the predictive performance of this model.

## Conclusion

In this study, we have developed the first prehospital predictive model with CHWs in LMICs to identify seriously ill children, ultimately demonstrating that the current standard of care has poor discrimination for health center admission. We also completed the first study evaluating CRP in the prehospital setting by CHWs and found that [1] CHWs can successfully administer advanced POCTs at the community level, and [2] CRP does not add predictive value for identifying children who will require health center admission. Finally, we found that a prehospital vital signs model is predictive for identifying children that require health center admission.

Future studies should have a larger sample size with greater socio-economic and geographic variability among villages. Prehospital and clinical data points should be compared for accuracy to determine agreement between CHWs and advanced health center providers. Additionally, a larger sample size would permit inclusion of other important outcomes such as early and late mortality. Currently, the LKP in Northern Uganda is preparing a larger prospective observational study, expanding from 4 to 30 CHWs, and will be well placed to address these issues. Given the success of this pilot study, subsequent expansion will include several other variables used in prediction scores in LMICs.

## References

[1] Vardell E. Global health observatory data repository. Med Ref Serv Q. 2020 Jan 2;39:67–9. doi: 10.1080/02763869.2019.1693231 32069199

[2] UNICEF. Levels and trends in child mortality. 2022.

[3] UDHS I. Uganda demographic and health survey. Kampala Uganda: Uganda Bureau of Statistics; 2011.

[4] Kane SS, Gerretsen B, Scherpbier R, et al. A realist synthesis of randomised control trials involving use of community health workers for delivering child health interventions in low and middle income countries. BMC Health Serv Res. 2010 Dec;10:1–7. doi: 10.1186/1472-6963-10-286 20942900 PMC2964693

[5] Mahmood H, Mckinstry B, Luz S, et al. Community health worker-based mobile health (mHealth) approaches for improving management and caregiver knowledge of common childhood infections: a systematic review. J Glob Health. 2020 Dec;10. doi: 10.7189/jogh.10.020438 PMC7774026 33437462

[6] Singh P, Sachs JD. 1 million community health workers in sub-Saharan Africa by 2015. Lancet. 2013 Jul 27;382:363–365. doi: 10.1016/S0140-6736(12)62002-9 23541538

[7] Boschi-Pinto C, Labadie G, Dilip TR, et al. Global implementation survey of integrated management of childhood illness (IMCI): 20 years on. BMJ Open. 2018 Jul 1;8:e019079. doi: 10.1136/bmjopen-2017-019079 PMC6067364 30061428

[8] Rowe AK, Rowe SY, Holloway KA, et al. Does shortening the training on integrated management of childhood illness guidelines reduce its effectiveness? A systematic review. Health Policy Plan. 2012 May 1;27:179–193. doi: 10.1093/heapol/czr033 21515912

[9] World Health Organization. Integrated management of childhood illness: distance learning course.

[10] Berg P, Lindhardt BØ. The role of procalcitonin in adult patients with community-acquired pneumonia—a systematic review. Dan Med J. 2012 Mar 1;59:A4357. 22381083

[11] Keitel K, Kagoro F, Samaka J, et al. A novel electronic algorithm using host biomarker point-of-care tests for the management of febrile illnesses in Tanzanian children (e-POCT): a randomized, controlled non-inferiority trial. PLOS Med. 2017 Oct 23;14:e1002411. doi: 10.1371/journal.pmed.1002411 29059253 PMC5653205

[12] Cook NR. Quantifying the added value of new biomarkers: how and how not. Diagn Progn Res. 2018 Jul 11;2:14. doi: 10.1186/s41512-018-0037-2 31093563 PMC6460632

[13] Christ-Crain M, Jaccard-Stolz D, Bingisser R, et al. Effect of procalcitonin-guided treatment on antibiotic use and outcome in lower respiratory tract infections: cluster-randomised, single-blinded intervention trial. Lancet. 2004 Feb 21;363:600–607. doi: 10.1016/S0140-6736(04)15591-8 14987884

[14] Tan M, Lu Y, Jiang H, et al. The diagnostic accuracy of procalcitonin and C‐reactive protein for sepsis: a systematic review and meta‐analysis. J Cell Biochem. 2019 Apr;120:5852–5859. doi: 10.1002/jcb.27870 30417415

[15] Leligdowicz A, Conroy AL, Hawkes M, et al. Risk-stratification of febrile African children at risk of sepsis using sTREM-1 as basis for a rapid triage test. Nat Commun. 2021 Nov 25;12:6832. doi: 10.1038/s41467-021-27215-6 34824252 PMC8617180

[16] Akech S, Kwambai T, Wiens MO, et al. Tackling post-discharge mortality in children living in LMICs to reduce child deaths. Lancet Child & Adolesc Health. 2023 Mar 1;7:149–151. doi: 10.1016/S2352-4642(22)00375-3 36682368

[17] Gupta MA, Chakrabarty A, Halstead R, et al. Validation of “signs of inflammation in children that kill” (SICK) score for immediate non-invasive assessment of severity of illness. Ital J Pediatr. 2010 Dec;36:1–6. doi: 10.1186/1824-7288-36-35 20420670 PMC2873401

[18] Kumar N, Thomas N, Singhal D, et al. Triage score for severity of illness. Indian Pediatr. 2003 Mar 1;40:204–210. 12657751

[19] Helbok R, Kendjo E, Issifou S, et al. The lambarene organ dysfunction score (LODS) is a simple clinical predictor of fatal malaria in African children. J Infect Dis. 2009 Dec 15;200:1834–1841. doi: 10.1086/648409 19911989

[20] Conroy AL, Hawkes M, Hayford K, et al. Prospective validation of pediatric disease severity scores to predict mortality in Ugandan children presenting with malaria and non-malaria febrile illness. Crit Care. 2015 Dec;19:1–1. doi: 10.1186/s13054-015-0773-4 25879892 PMC4339236

[21] Moons KG, Altman DG, Reitsma JB, Ioannidis JP, Macaskill P, Steyerberg EW, et al. Transparent reporting of a multivariable prediction model for individual prognosis or diagnosis (TRIPOD): explanation and elaboration. Ann Intern Med. 2015 Jan 6;162:W1–73. doi: 10.7326/M14-0698 25560730

[22] Ebbs D, Benson O, Jasicki S, et al. The Laro Kwo project: a train the trainer model combined with mobile health technology for community health workers in Northern Uganda. PLOS Global Public Health. 2023 May 17;3:e0001290. doi: 10.1371/journal.pgph.0001290 37195969 PMC10191267

[23] Goldstein B, Giroir B, Randolph A. International pediatric sepsis consensus conference: definitions for sepsis and organ dysfunction in pediatrics. Pediatr Crit Care Med. 2005 Jan 1;6:2–8. doi: 10.1097/01.PCC.0000149131.72248.E6 15636651

[24] Riley RD, Ensor J, Snell KI, et al. Calculating the sample size required for developing a clinical prediction model. BMJ. 2020 Mar 18;368:m441. doi: 10.1136/bmj.m441 32188600

[25] Moons KG, Donders RA, Stijnen T, et al. Using the outcome for imputation of missing predictor values was preferred. J Clin Epidemiol. 2006 Oct 1;59:1092–1101. doi: 10.1016/j.jclinepi.2006.01.009 16980150

[26] Robin X, Turck N, Hainard A, et al. pROC: an open-source package for R and S+ to analyze and compare ROC curves. BMC Bioinformatics. 2011 Dec;12:1–8. doi: 10.1186/1471-2105-12-77 21414208 PMC3068975

[27] DeLong ER, DeLong DM, Clarke-Pearson DL. Comparing the areas under two or more correlated receiver operating characteristic curves: a nonparametric approach. Biometrics. 1988 Sep;1:837–845. doi: 10.2307/2531595 3203132

[28] Loader C. Smoothing: local regression techniques. Handb Comput Stat Concepts Methods. 2012;1:571–596.

[29] Team RD. R: a language and environment for statistical computing. 2010.

[30] Chandna A, Lubell Y, Mwandigha L, et al. Defining the role of host biomarkers in the diagnosis and prognosis of the severity of childhood pneumonia: a prospective cohort study. Sci Rep. 2023 Jul 25;13:12024. doi: 10.1038/s41598-023-38731-4 37491541 PMC10368669

